# Journalism in the South

**Published:** 1882-06-20

**Authors:** 


### *JOURNALISM IN THE SOUTH.*

In former times the rule was failure in respect to all efforts of medical journalism in the Southern States, and even now the difficulties of the Southern journalist are far greater than in the Northern States. It may well be asked why is this the case? To this question we have to make the humiliating answer that a great many practitioners do not take the journals, and do not read or encourage the medical literature of their section. And the majority of those who do read take the Northern journals in preference to Southern journals, under the mistaken impression, perhaps, that they are better, or frequently because they are cheaper. The duty of sustaining the literature of their own section is disregarded, and the important fact that the diseases and the practice at home are different does not seem to be considered. While this is true of the Southern physicians, the reverse has not been the case with Northern physicians. Southern doctors take Northern journals, but Northern doctors do not take Southern journals.

We are glad to report some recent indications of improvement upon the points mentioned—a growing appreciation of the ability and practical features of the Southern journals and of their superior adaptation to the wants of the Southern practitioner. While an occasional new subscriber from the North shows that even they are beginning to discover that something good may come out of Nazareth. Let the friends of the RECORD stand by us. Let them write for us, sending short, interesting and practical articles; and let them use their influence in extending our circulation, that our journal may, in the future, as in the past, maintain a growing and constantly increasing interest and usefulness as an exponent and representative of medical literature in the South.

